# Identification and validation of FPR1, FPR2, IL17RA and TLR7 as immunogenic cell death related genes in osteoarthritis

**DOI:** 10.1038/s41598-023-43440-z

**Published:** 2023-10-06

**Authors:** Tingting Ren, Nuo Yin, Li Du, Mingmang Pan, Liang Ding

**Affiliations:** 1 Department of Critical Care Medicine, Renji Hospital, School of Medicine, Shanghai Jiaotong University, Shanghai, 200127 China; 2 Department of Orthopaedics, Shanghai Jiao Tong University Affiliated Sixth People’s Hospital South Campus, Shanghai, 201400 China

**Keywords:** Immunological disorders, Genetics, Immunology, Diseases, Pathogenesis

## Abstract

Immunogenic cell death (ICDs) has gained increasing attention for its significant clinical efficacy in various diseases. Similarly, more and more attention has been paid in the role of immune factors in the pathological process of osteoarthritis (OA). The objective of this study is to reveal the relationship between ICD-related genes and the process of OA at the gene level through bioinformatics analysis. In this study, Limma R package was applied to identify differentially expressed genes (DEG), and OA related module genes were determined by weighted gene co-expression network analysis. The ICD-related genes were extracted from a previous study. The module genes related to DEGs and ICD were overlapped. Then, hub genes were identified by a series of analyses using the Least absolute shrinkage and selection operator and random forest algorithm, the expression level and diagnostic value of hub genes were evaluated by Logistic regression. In addition, we used Spearman rank correlation analysis to clarify the relationship between hub genes and infiltrating immune cells and immune pathways. The expression levels of FPR1, FPR2, IL17RA, and TLR7 was verified in SD rat knee joint model of OA by immunohistochemistry. The expression levels of FPR1, FPR2, IL17RA, and TLR7 mRNA were detected in the IL-1β induced rat chondrocytes in qPCR experiment in vitro. Four hub genes (FPR1, FPR2, IL17RA, and TLR7) were ultimately identified as OA biomarkers associated with ICD. And knockdown of TLR7 reversed collagen II and ADAMTS-5 degradation in IL-1β-stimulated chondrocytes. This research may provide new immune related biomarkers for the diagnosis of OA and serve as a reference for disease treatment monitoring.

## Introduction

Immunogenic cell death (ICD) is a form of regulated cell death (RCD) usually associated with malignant and infectious diseases^1–4^. However, increasing evidence shows that normal cells may trigger antigen-specific immune responses in specific lesions, suggesting that ICDs may also play a role in other kinds of diseases^5^.

Although the pathogenesis of osteoarthritis (OA) remains unclear, studies have shown that synovitis accompanied by inflammatory and abnormal immune responses damage are detected in OA^6,7^. The immunological disorders will affect the chondrocytes proliferation and cartilage matrix deposition, thus lead to an imbalance between the synthesis and decomposition of chondrocytes^8^. The most common resident and infiltrating immune cells types in OA are macrophages, T cells and mast cells (MCs)^9^, suggesting that immune factors are critical in OA. However, the immune mechanisms and ICD associated processes of OA have not been clarified, emphasizing the valuation to explore the ICD genes related to OA.

The purpose of this study is to identify the genes related to ICD and OA, provide reference for the target of OA treatment. These identified genes can also be used as biomarkers for diagnosis and treating monitors of OA.

## Materials and methods

### Data preprocessing and study design

The gene expression levels of normal and OA samples were downloaded from the Gene Expression Omnibus (GEO) database (https://www.ncbi.nlm.nih.gov/geo/), which includes two data sets, GSE51588 and GSE129147. There were 50 OA and 19 normal samples selected for analysis from GSE55235 and GSE55457 datasets in total. The R “limma” and “ggplot2” software packages were applied to process gene expression data and visualize the results^10^. The t-test was used to screen. Genes were considered as differentially expressed genes (DEGs) when the *P* values < 0.05 (BH method) and |log2FC| > 0.5^11–13^. Volcano plots were drawn by “ggplot2” and “ggrepel” R packages. A heat map was drawn by “pheatmap” and “dplyr” R packages.

### Weighted gene co-expression network analysis (WGCNA)

The “WGCNA” R package was applied to construct the gene co-expression network and search for clinical trait related modules and hub genes^14^. Genes were divided into different gene modules based on different topological overlap matrix (TOM)-based dissimilarity measure. The hierarchical clustering method was used to identify the modules and calculate the characteristic genes. And modules whose correlations of module eigengenes exceeded a threshold of 0.75 were merged. After that, we used the Pearson correlation analysis to evaluate the correlation between phenotypes (OA or control samples) and each module. Finally the OA related modules were identified. The module which had the highest correlation with clinical traits was selected as the hub genes. Hub genes were defined as gene significance (GS) > 0.5 and module membership (MM) > 0.6.

### Gene-ontology (GO) and kyoto encyclopedia of genes and genomes (KEGG) pathway enrichment analysis of DEGs and overlapped genes

ICD-related genes were downloaded from Garg AD’s study^15^, and these genes were overlapped with DEGs and OA-related module genes derived from WGCNA. We used the Venn diagram to describe the details of the overlapped genes. Enrichment analysis of GO and KEGG pathways^16^ was performed to identify the pathways of genes, and the results were visualized using “limma”, “org.hs.eg. db”, “clusterProfiler” and “Enrichment Plot” R packages^17^.

### Feature selection by least absolute shrinkage and selection operator (LASSO) and random forest algorithm

Univariate regression analysis was performed on the overlapping genes obtained by single sample gene set enrichment analysis (ssGSEA) to screen genes. LASSO logistic regression^18^ and Random Forest algorithm^19^ were used to reduce the number of prognostic genes.

### Construction and validation of the logistic regression

The logistic regression model and receiver operating characteristic (ROC) curve analysis were performed by the R “pROC” package to distinguish OA patients from the control group^20^. The statistically significant genes from the hub genes were selected (*P* < 0.05), and the occurrence of OA was predicted by the nomogram. The expression level of the hub gene was indicated by the violin plot.

### Identification of the correlation between hub genes expression and immune infiltration and functional pathway of OA

According to the expression levels of immune cell-specific marker genes, the immune infiltration and functional pathways were calculated by ssGSEA scores^21^. We obtained 28 types of immune cells from a previous article^22^. The R “GSVA” package^21^ for ssGSEA was applied to obtain enrichment scores for each immune-related term. DEGs of OA and normal samples were analyzed using the “vioplot” package of R language. The results were then visualized using the R “pheatmap” software package. The R package “ggplot2” and “reshape2” were used for Spearman rank correlation analysis (Supplementary Information 1).

### Immunohistochemistry (IHC) and image analysis

Adult male Sprague Dawley (SD) rats (10 weeks old, weighing 225–250 g) from Jihui Laboratory Animal Care Co., Ltd (Shanghai, China) were used in this study. OA model (MMT) was induced as described previously^23^. A total of 18 animals were randomly divided into two groups (control, n = 8; MMT group, n = 10). All animal experiments were approved by the Animal Care and Use Committee of Shanghai Jiao Tong University Affiliated Sixth People’s Hospital South Campus, in accordance with the Guidelines for the Care and Use of Laboratory Animals (National Institutes of Health, Bethesda, MD, USA).The tissue preparation of the entire knee joint and Immunostaining were performed as previously described^24^. For immunohistochemistry, the primary antibodies (Abs) were FPR1 Ab (1:100; Zenbio, Chengdu, China, 862749), FPR2 Ab (1:100; Santa Cruz, CA, USA, #sc-32266), IL17RA Ab (1:500; Servicebio, Wuhan, China, GB11110), and TLR7 Ab (1:200; Servicebio, Wuhan, China, GB112633). Secondary Abs were goat anti-mouse/rabbit IgG polymer antibody. Three different areas were randomly selected from the high-power image (20× magnification) for counting. The percentage of positivity of each immunohistochemical assay was measured and quantificated using the Image-Pro Plus (U.S. MEDIA CYBERNETICS) software.

### Cell culture and reagents

According to the previous study^25^, rat primary chondrocytes were isolated from the knee articular cartilage of 4-week-old SD rats. The cells were cultured in RPMI-1640 with 10% fetal bovine serum (abs972, Absin Bioscience Inc., Shanghai, China), 1% penicillin and 100 µg/ml streptomycin (Hyclone Laboratories Inc., Logan, UT, United States) at 37 °C in a 5% CO2 incubator.

### RNA interference

Chemically modified small interfering RNAs (siRNAs) targeting TLR7 and negative control RNAs (siCont) were purchased from RiboBio Company (Guangzhou, China). RiboFECT™ CP Reagent (RiboBio, Guangzhou, China) was used to transfect siRNAs according to the manufacturer’s instructions.

### RNA extraction and RT-qPCR

Interleukin-1β (IL-1β) can induce cartilage degradation in chondrocytes, which is widely used in chondrocytes as an induction model for osteoarthritis^26,27^. We stimulated rat chondrocytes with IL-1β and verified the expression level of hub gene using qRT-PCR. Chondrocytes were added to the normal medium (NC group) or treated with IL-1β (10 ng/mL) culture medium (IL-1β group). After incubation for 48 h, the mRNA relative expression of FPR1, FPR2, IL17RA and TLR7 were detected. Total RNA was extracted from primary rat chondrocytes using the TRIzol Reagent (Biosharp, Hefei, Anhui, China). Next, RNA was reverse transcribed into cDNA using the Vazyme HiScriptIIQ RT SuperMix (KCD-M1003, Cronda, Beijing, China) for qPCR following the manufacturer’s instructions. Gene expressional levels were finally determined using the real-time quantitative PCR method with the 2 × Q3 SYBR qPCR Master mix (Universal) (KCD-M1004, Cronda, Beijing, China) according to the manufacturer’s instructions. PCR conditions were as follows: step 1, 95 °C for 2 min; step 2, 95 °C for 10 s, and 45 cycles at 60 °C for 20 s; step 3, 95 °C for 15 s, 60 °C for 60 s, and 40 °C for 10 s. According to the previous study^28^, the relative mRNA expression was calculated using the 2−ΔΔCq method, and the values were expressed based on the fold-change relative to β-actin. The target gene primers were designed and purchased from Hasenbio, China (Table 1), and β-actin was used as the control.

**Table 1:** PCR primer sequences (rat).

Name		Sequence
FPR1	Forward	5′-TGGAGTCTTGGGCAACGG-3′
	Reverse	5′-CATGACCAGGCTGACGATG-3′
FPR1	Forward	5′-AAGTGCTGGACGTAGCAAAC-3′
	Reverse	5′-AAGGGAAACCAACAGATAAAGA-3′
IL17RA	Forward	5′-GCAGAAGCAGGAAATGGAGGAG-3′
	Reverse	5′-CAAACAATGTAGGTGCCGAAGC-3′
TLR7	Forward	5′-CAACTGTCCCTGCGAGAT-3′
	Reverse	5′-GCAAAGAAAGCGATTGTGAT-3′
β-actin	Forward	5′-CACCCGCGAGTACAACCTTC-3′
	Reverse	5′-CCCATACCCACCATCACACC-3′

### Western blot analysis and antibodies

Western blots were performed as described^29^ with primary antibodies targeting Collagen II (1:100, #BA0533, BOSTER, China) and ADAMTS-5 ((A Disintegrin and Metalloproteinase with Thrombospondin motifs-5) (1:1000, DF13268, Affnity, China). Antibody targeting β-actin (1:5000, 81115-1-RR, Proteintech, China) was used as a control. The membranes were incubated at 25℃ in the secondary antibody for 2 h. After washing, we used ECL kit (P0018, Beyotime, China) to detect the signal.

### Statistical analysis

Data were expressed as means ± SD and analyzed using SPSS 19.0 software (IBM Corp, Armonk, NY). Student’s t test was used for comparisons between the two groups. A *P* value of less than 0.05 was considered statistically significant.

### Ethics approval and consent to participate

The animals used in this study were treated strictly according to the Animal (Scientific Procedures) Act 1986. This study was approved by the Institutional Review Board of Shanghai Jiao Tong University Affiliated Sixth People’s Hospital South Campus. Moreover, this study is reported in accordance with ARRIVE guidelines (https://arriveguidelines.org/).

## Results

### DEGS in OA and normal samples

**Figure 1:**
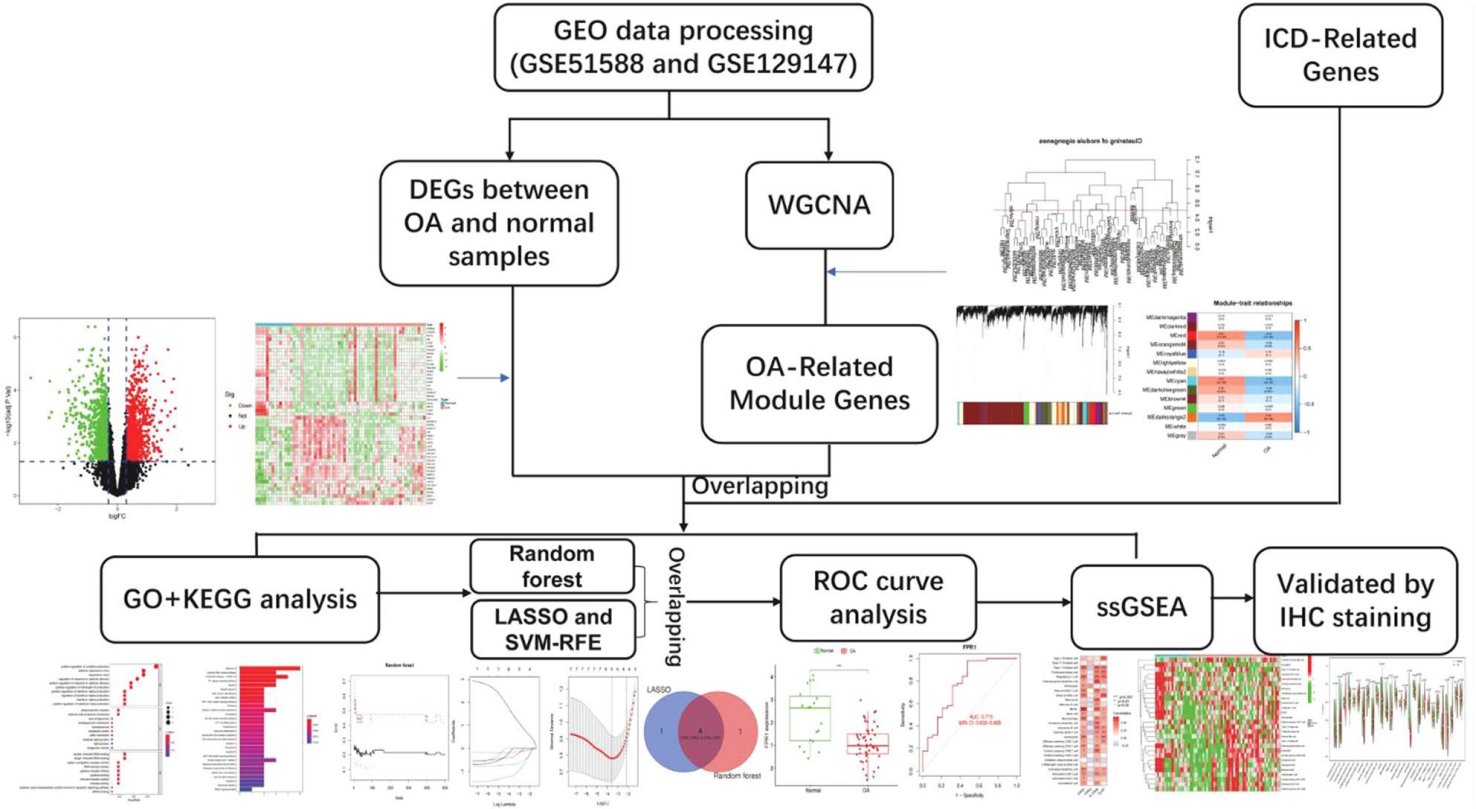
The workflow of the study.

**Figure 2:**
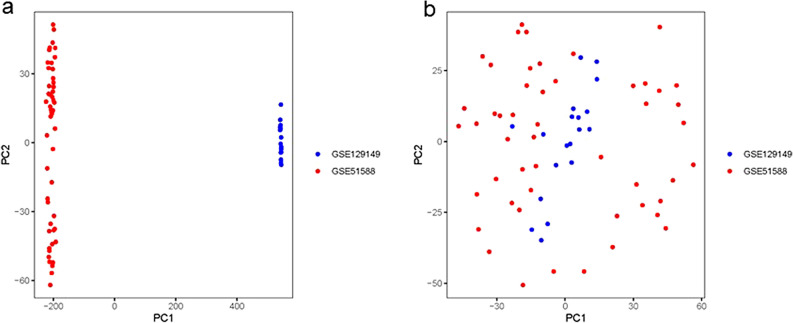
Data preprocessing. Data in GSE51588 and GSE129149 was normalized using combat procedure. The PCA before (**a**) and after (**b**) normalized was presented.

**Figure 3:**
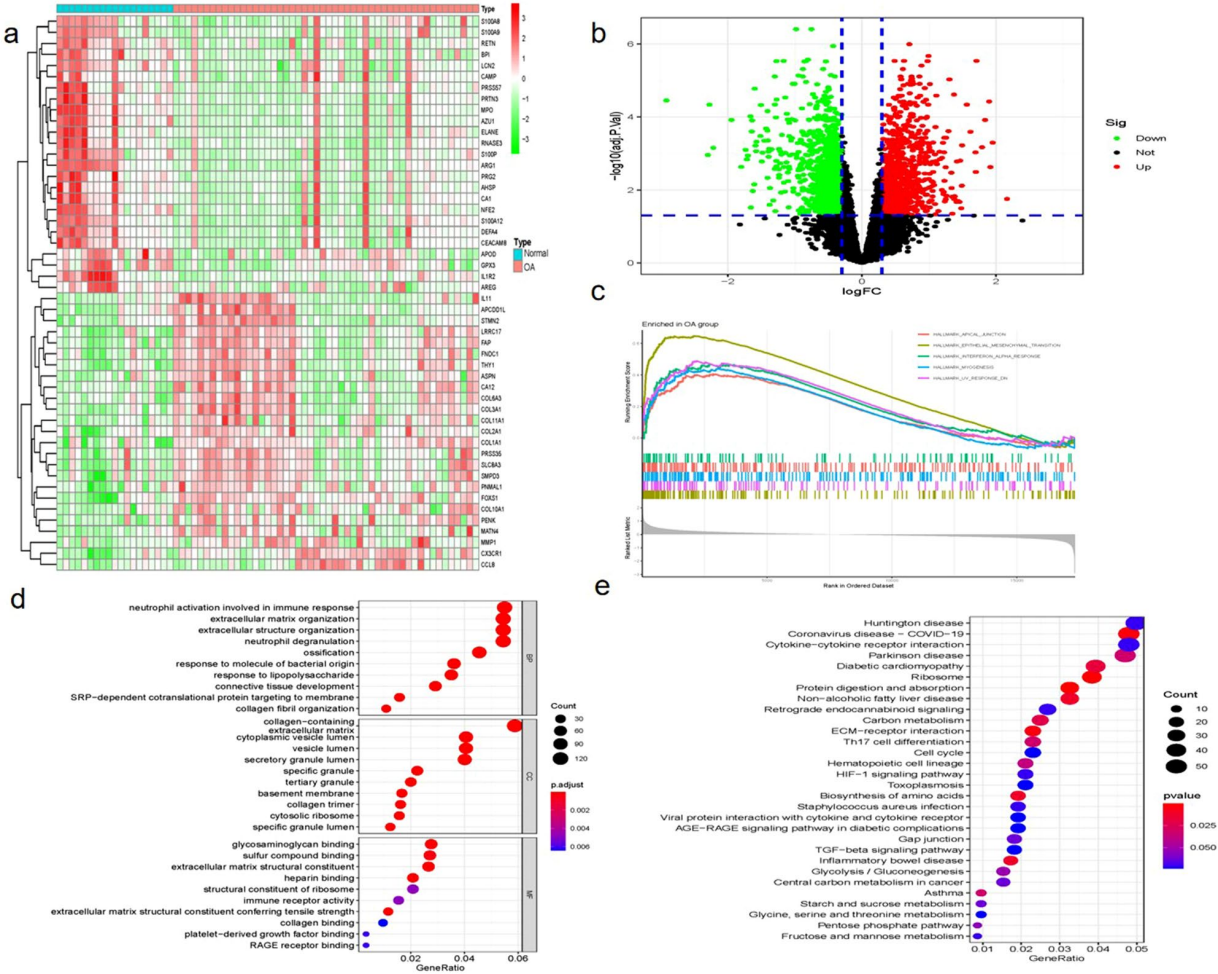
DEGs in OA and normal Samples. The composition of top 50 DEGs (25 upregulated and 25 downregulated) was showed in a heatmap (**a**) and Volcano plots (**b**). Red: upregulated expression; green: downregulated expression. GSEA enrichment analysis results (**c**). GO functional analysis results (**d**). KEGG enrichment analysis results (**e**). Red: upregulated expression; blue: downregulated expression.

Two microarray raw datasets (GSE51588 and GSE129147), including 50 OA and 19 normal samples (Supplementary Figures 1, 2), were selected for the study of immune cell infiltration (Fig. 1). The data before (a) and after (b) PCA was presented in Fig. 2. In our study, 2313 differentially expressed mRNAs were identified. The top 50 differentially expressed mRNAs (25 upregulated and 25 downregulated) were respectively demonstrated by heatmapping (Fig. 3a) and Volcano plots (Fig. 3b). Figure 3c shows the results of GSEA enrichment analysis. Figure 3d shows the GO terms enrichment analysis results. Figure 3e shows the results of KEGG enrichment analysis.

### Weighted co-expression network construction and identification of core modules

**Figure 4:**
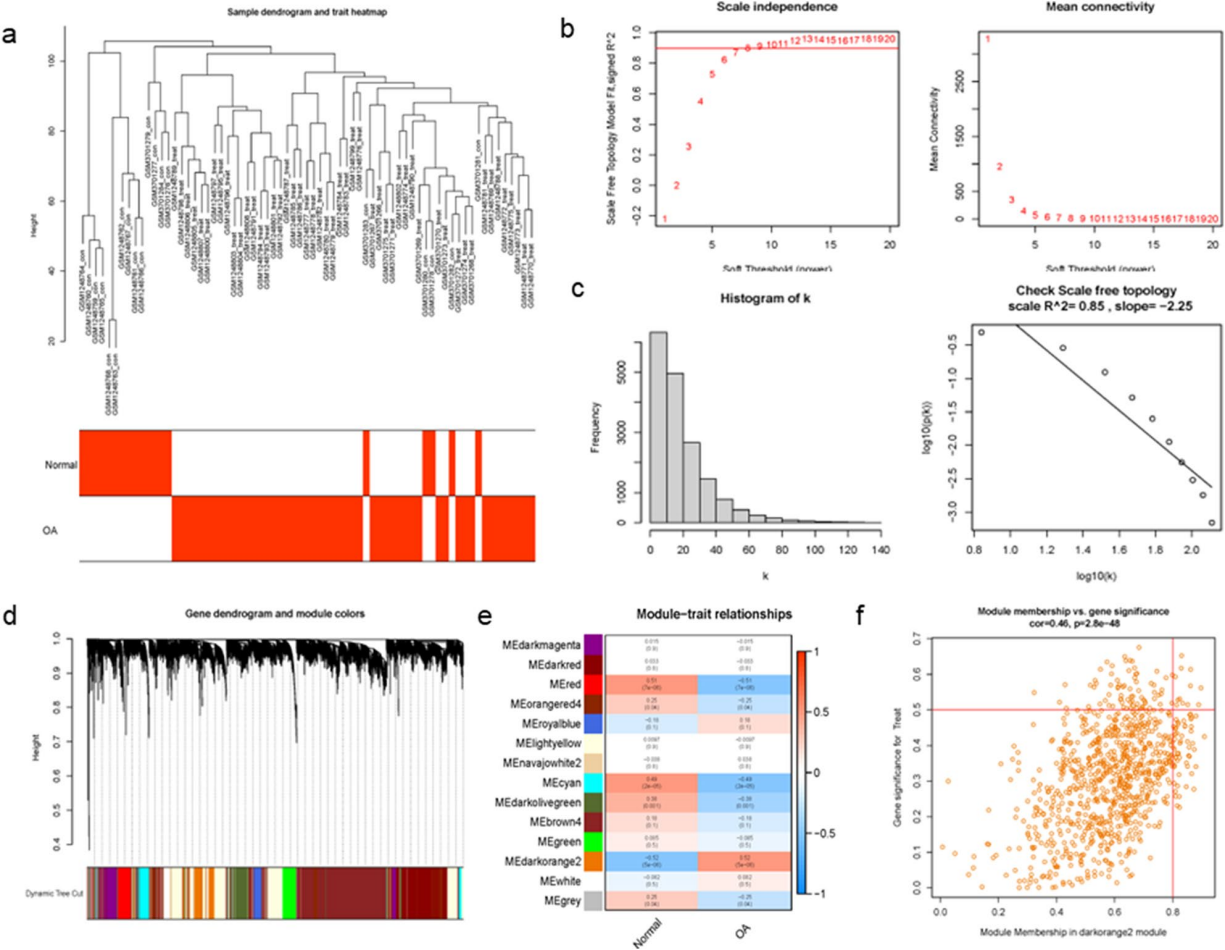
Construction of WGCNA modules. (**a**) Clustering dendrogram and trait heatmap of samples (Red indicates the RA group and white indicates the Normal group. (**b**) The soft thresholding and correlation coefficient in the scale-free topology fitting graph. (**c**) Histogram of the connectivity distribution and the scale-free topology. (**d**) The dendrogram of differentially expressed genes (Clustering based on dissimilarity measures). (**e**) Module and trait-related heat map. (**f**) A scatterplot of Gene Significance (GS) for weight versus Module Membership (MM) in the module darkorange2. There is a highly significant correlation between GS and MM in this module.

After the average linkage hierarchical clustering, 14 modules were identified. Sample clustering dendrogram and trait heatmap based on Euclidean distance. A scale-free network was built, and the soft threshold was set to 4 (R^2^ = 0.85). We established adjacency matrix and topological overlap matrix, and calculate module characteristic genes. Then, we clustered them according to the correlation and analyzed the correlation between each characteristic gene and phenotype (OA or control sample). We found that darkorange2 (Cor = 0.52, *P* value = 5E−06) module had the highest correlation with OA (Fig. 4). Among these modules, a total of 4100 OA-related genes were retained for further analysis (Supplementary Figures 3–7).

### Overlap OA disease-related module genes with ICD-related genes and OA DEGs

**Figure 5:**
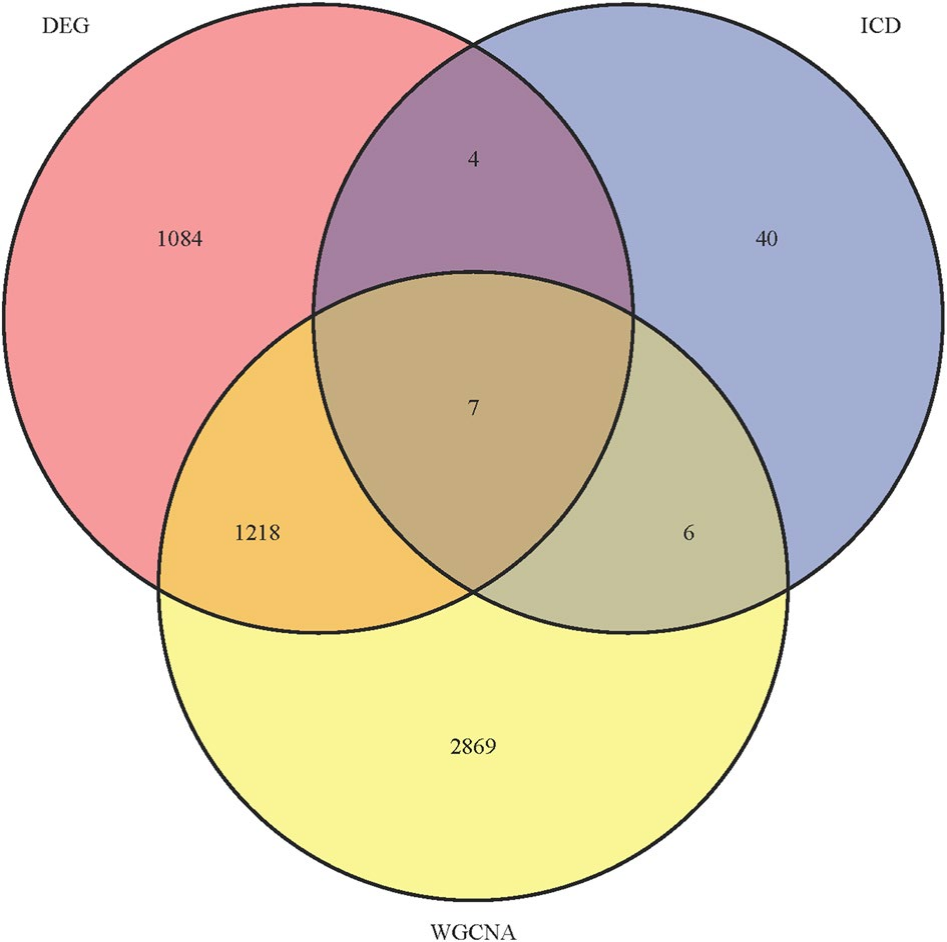
Venn diagram shows seven Overlapped genes.

We extracted 57 ICD-related genes from the work of Garg AD et al.^15^. After the OA-related module genes of WGCNA were overlapped with ICD-related genes and OA DEGs, 7 overlapping genes were obtained, as shown in Fig. 5.

### GO and KEGG enrichment analysis of overlapped genes

**Figure 6:**
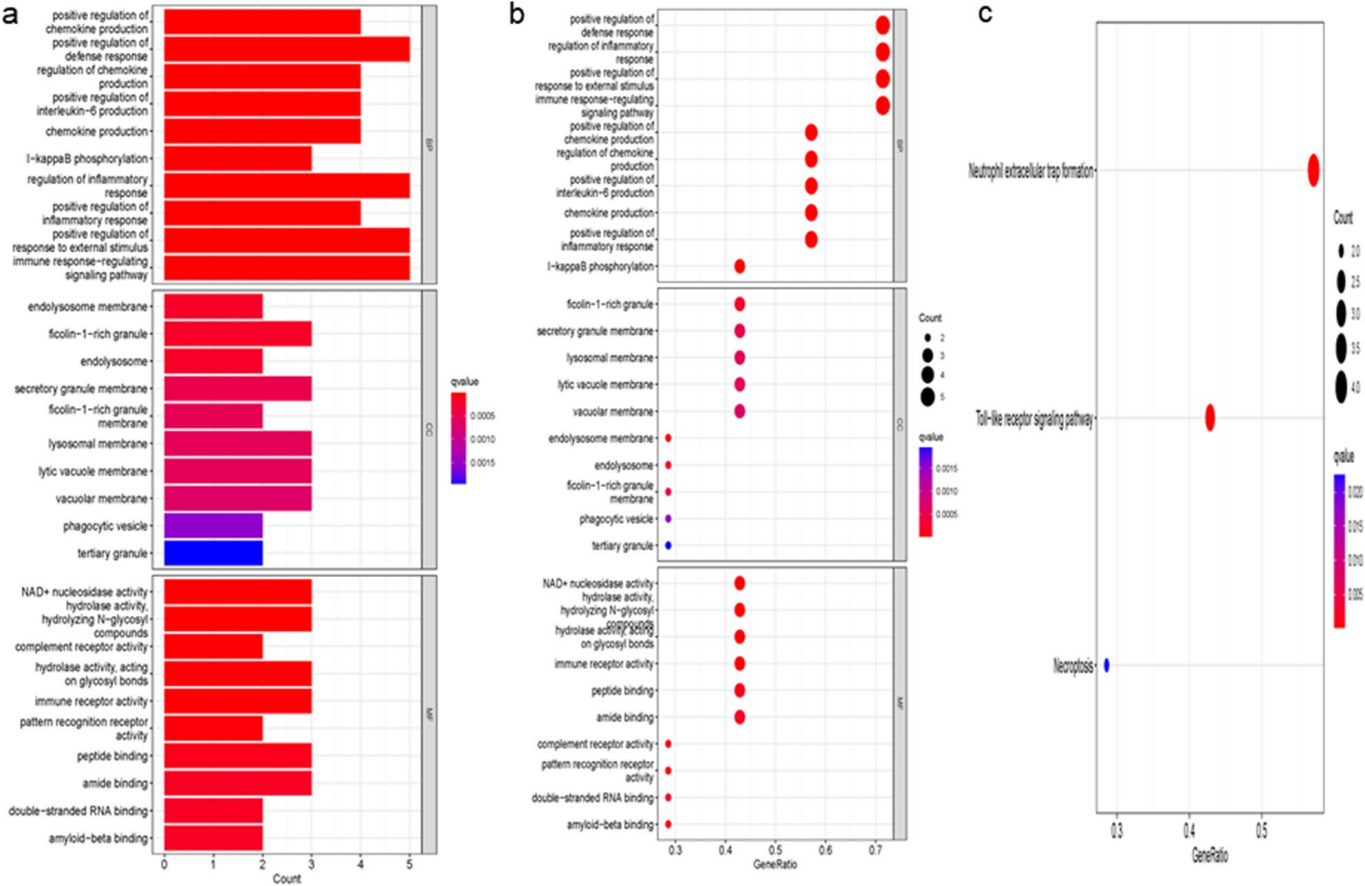
GO and KEGG analysis. GO functional analysis by bar plot (**a**) and y bubble (**b**). KEGG pathway enrichment analysis of hub genes (**c**).

**Figure 7:**
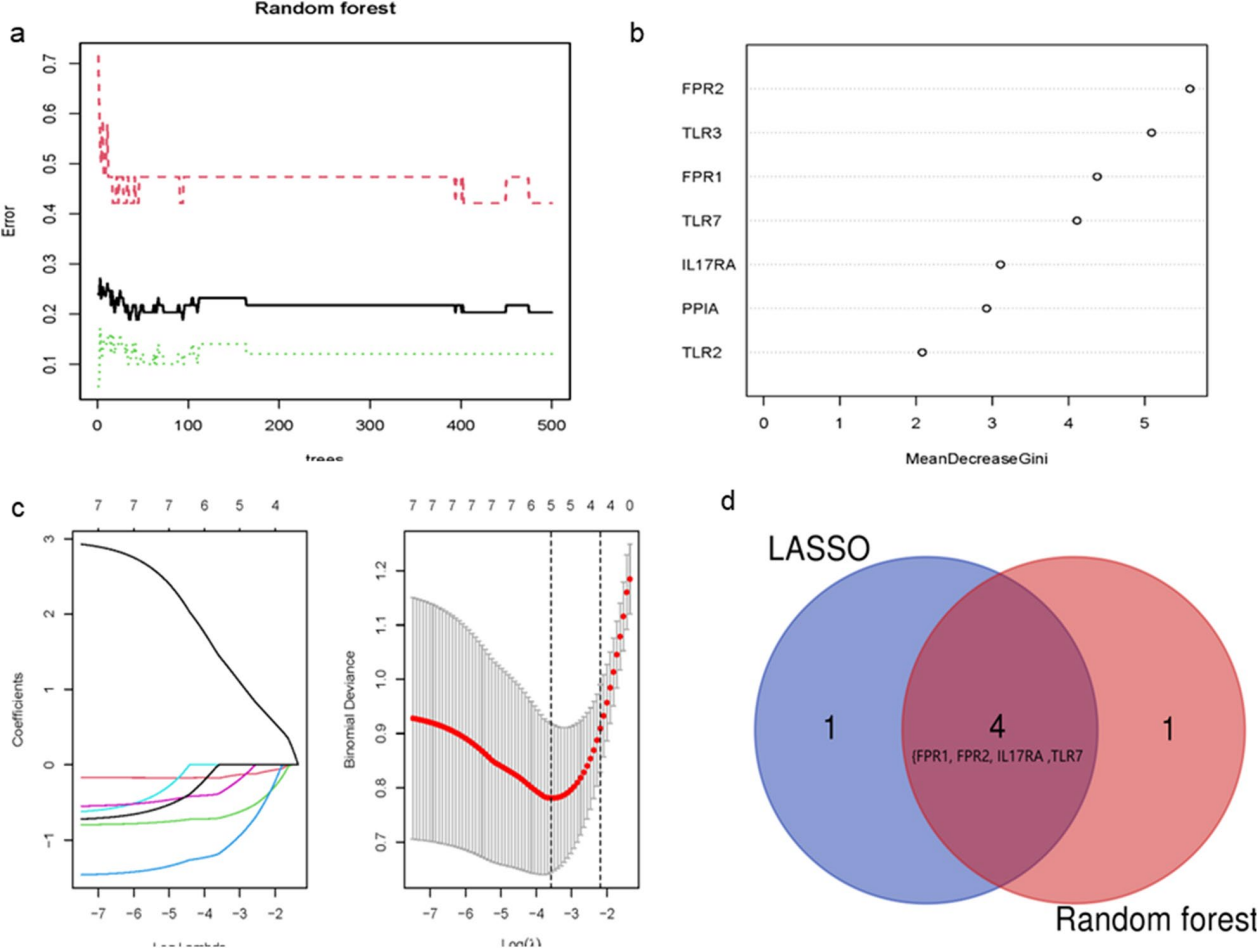
Random Forest and LASSO were used for feature selection. The error in random forest algorithm (**a**) Characteristic gene Gini index (**b**). (**c**) LASSO was conducted for biomarkers screening. (**d**) Overlapped the Random Forest and LASSO, there were 4 genes (FPR1, FPR2, IL17RA and TLR7) identified.

The GO functional terms of seven ICD-related OA genes were shown in Fig. 6. Five genes were identified by LASSO and SVM algorithms respectively. After overlapping, we obtained four biomarkers: formylpeptide receptor 1(FPR1), formylpeptide receptor 2(FPR2), interleukin-17 receptor A (IL17RA), and Toll-like receptor 7(TLR7), which were associated with the prognosis of OA (Fig. 7).

### Identification of hub gene expression levels and diagnostic value

**Figure 8:**
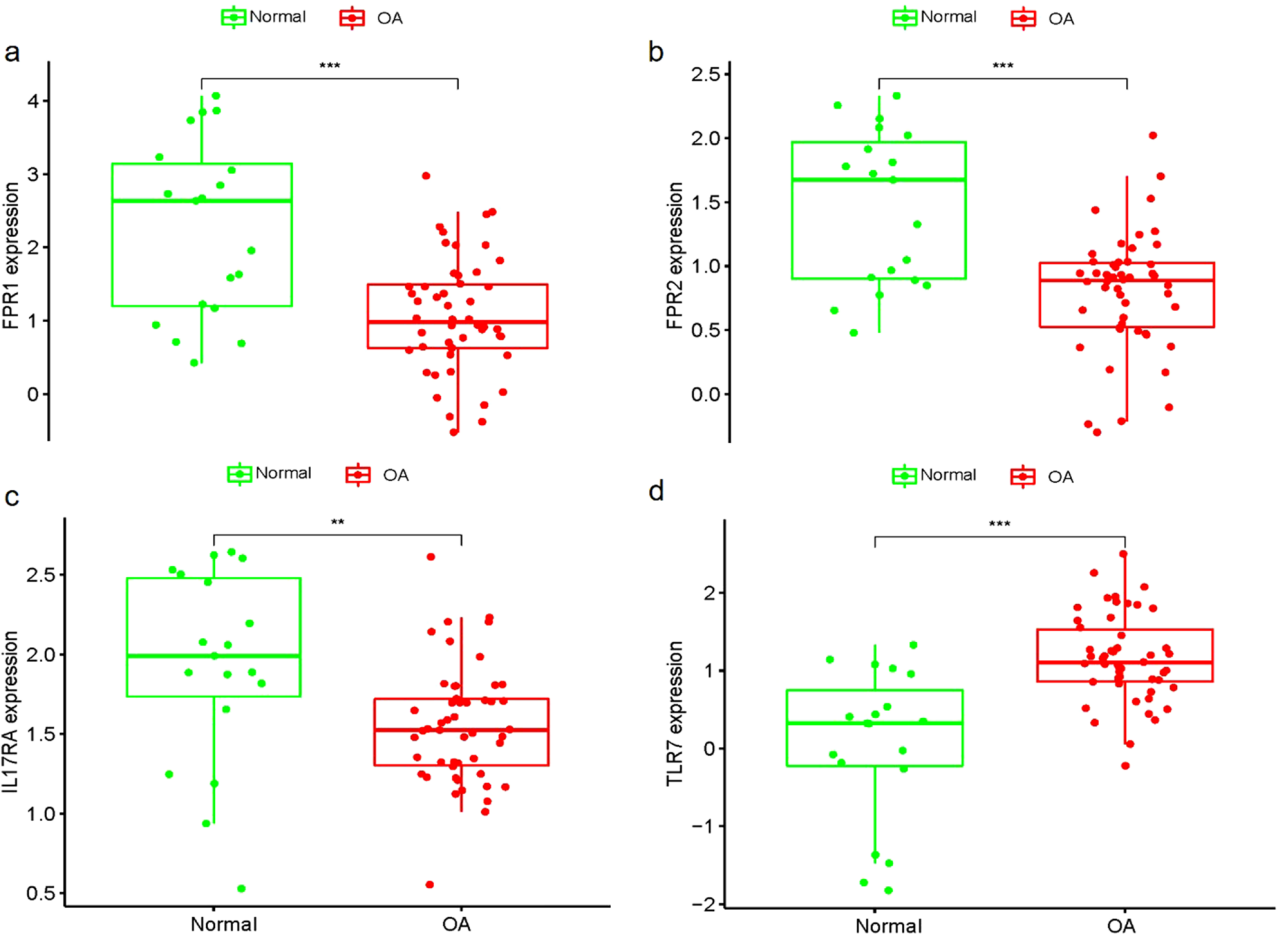
Validation of hub genes in the gene expression level. FPR1 (**a**), FPR2 (**b**) and IL17RA (**c**) were significantly lower expression in OA samples compared with healthy controls, while TLR7 (**d**) were significantly higher expression in OA samples compared with healthy controls. ***P < 0.001, **P < 0.01.

**Figure 9:**
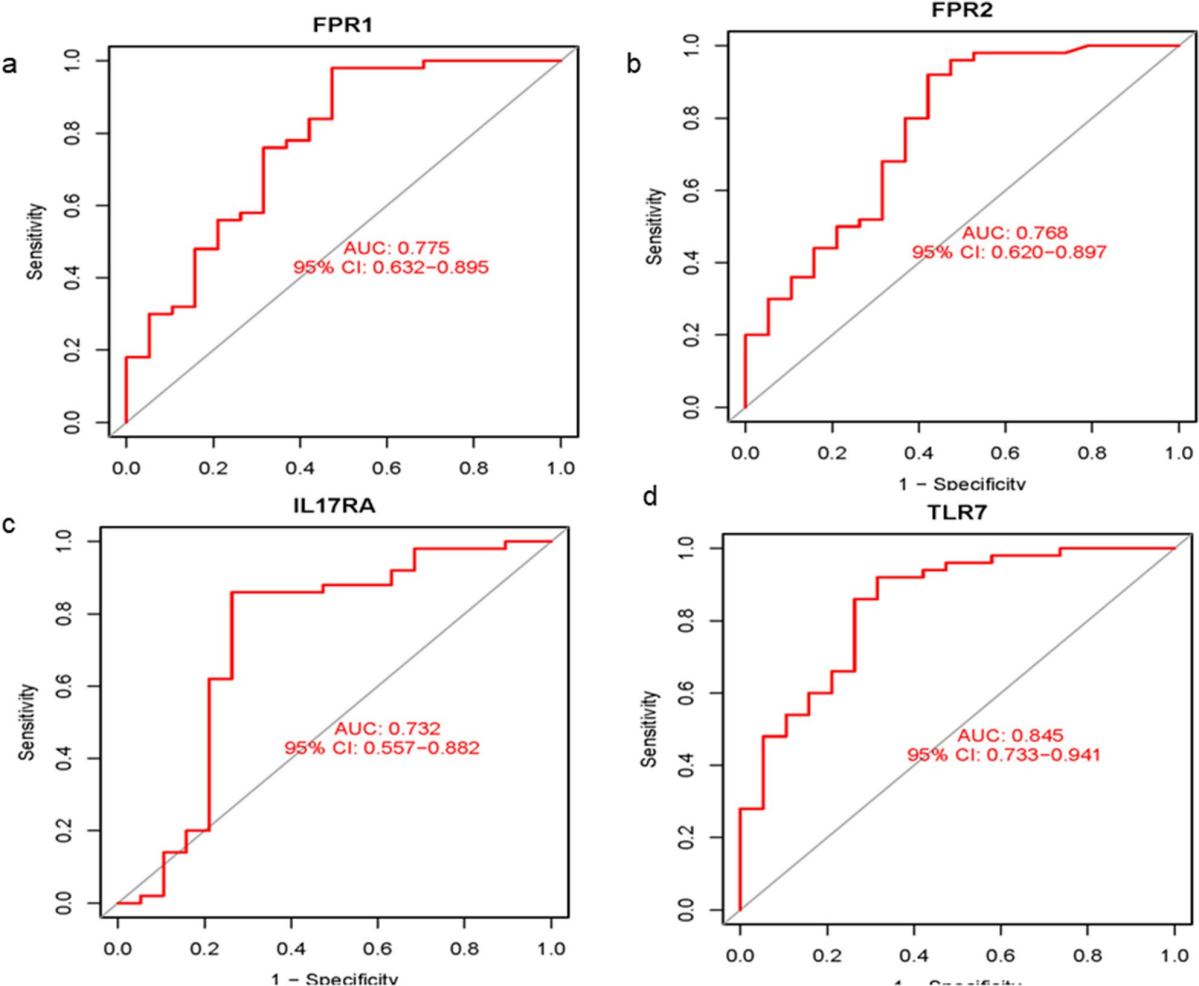
ROC curve and AUC statistic of the four specifically expressed hub genes in OA and healthy samples.

The box plots were used to validate the expression levels of the four hub genes. Figure 8 demonstrates FPR1 (*P* < 0.001), FPR2 (*P* < 0.001) and IL17RA (*P* < 0.01) had significantly lower expression in OA samples compared with healthy controls, while TLR7 (*P* < 0.001) showed significantly higher expression in OA samples compared with healthy controls. We used ROC curve and nomograph to evaluate the logistic regression model and predict the occurrence of OA respectively (Fig. 8). The ROC curve results showed that the area under the curve (AUC) values of all four hub genes were > 0.73, which indicated that these genes had diagnostic value for OA (Fig. 9).

### Immune cell infiltration and immune functional differences with its correlation of hub genes

**Figure 10:**
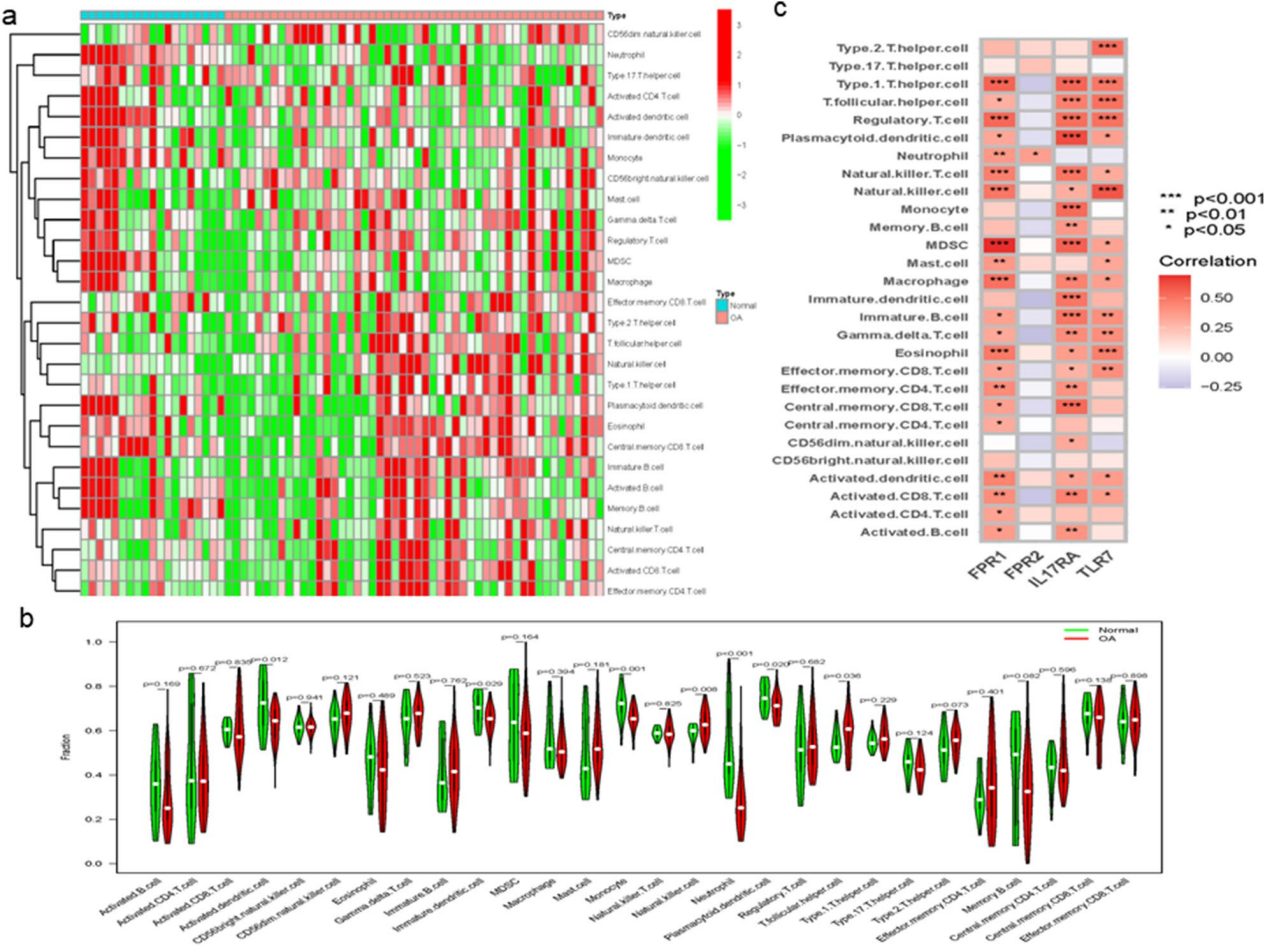
Analysis of immune landscape associated with OA. Heatmap (**a**) and violin plot (**b**) showing the distribution of 28 types of immune cells in healthy control and OA samples. (**c**) The relationship between four hub genes and immune cell infiltration.

The ssGSEA algorithm was used to evaluate the differences in immune cell infiltration between OA and healthy controls. Figure 10 shows the distribution of 28 immune cells in samples. The results of immunocyte infiltration analysis showed that the infiltration of activated dendritic cells, immature dendritic cells, Monocyte, Natural killer cells, Neutrophi, Plasmacytoid dendritic cells and T follicular helper cells were significantly higher in OA than in healthy samples, suggesting that these cells played an important role in the development of OA (Fig. 10). The correlation analysis showed that Type1 T helper cell (*P* < 0.001), T follicular helper cell (*P* < 0.05) , Regulatory T cell (*P* < 0.001), Plasmacytoid dendritic cell (*P* < 0.05), Neutrophil (*P* < 0.01), Natural killer T cell (*P* < 0.001), Natural killer cell (*P* < 0.001), MDSC (*P* < 0.001), Mast cell (*P* < 0.01), Macrophage (*P* < 0.001), Immature B cell (*P* < 0.05), Gamma delta T cell (*P* < 0.05), Eosinophil (*P* < 0.001), Effector memory CD8 T cell (*P* < 0.05), Effector memory CD4 T cell (*P* < 0.01), Central memory CD8 T cell (*P* < 0.05), Central memory CD4 T cell (*P* < 0.05), Activated dendritic cell (*P* < 0.01), Activated CD8 T cell (*P* < 0.01), Activated CD4 T cell (*P* < 0.05) and Activated B cell (*P* < 0.05) were positively correlated with FPR1. Neutrophil (*P* < 0.05) was positively correlated with FPR2. Type 1 T helper cell (*P* < 0.001), T follicular helper cell (*P* < 0.001), Regulatory T cell (*P* < 0.001), Plasmacytoid dendritic cell (*P* < 0.001), Natural killer T cell (*P* < 0.001), Natural killer cell (*P* < 0.05), Monocyte (*P* < 0.001), Memory B cell (*P* < 0.01), MDSC (*P* < 0.001), Macrophage (*P* < 0.01), Immature dendritic cell (*P* < 0.001), Immature B cell (*P* < 0.001), Gamma delta T cell (*P* < 0.01), Eosinophil (*P* < 0.05), Effector memory CD8 T cell (*P* < 0.05), Effector memory CD4 T cell (*P* < 0.01), Central memory CD8 T cell (*P* < 0.001), CD56dim natural killer cell (*P* < 0.05), Activated dendritic cell (*P* < 0.05), Activated CD8 T cell (*P* < 0.01), Activated B cell (*P* < 0.01) were positively correlated with IL17RA. Type 2 T helper cell (*P* < 0.001), Type 1 T helper cell (*P* < 0.001), T follicular helper cell (*P* < 0.001), Regulatory T cell (*P* < 0.001), Plasmacytoid dendritic cell (*P* < 0.05), Natural killer T cell (*P* < 0.05), Natural killer cell (*P* < 0.001), MDSC (*P* < 0.05), Mast cell (*P* < 0.05), Macrophage (*P* < 0.05), Immature B cell (*P* < 0.01), Gamma delta T cell (*P* < 0.01), Eosinophil (*P* < 0.01), Effector memory CD8 T cell (*P* < 0.01), Activated dendritic cell (*P* < 0.05) and Activated CD8 T cell (*P* < 0.05) were positively correlated with TLR7.

**Figure 11:**
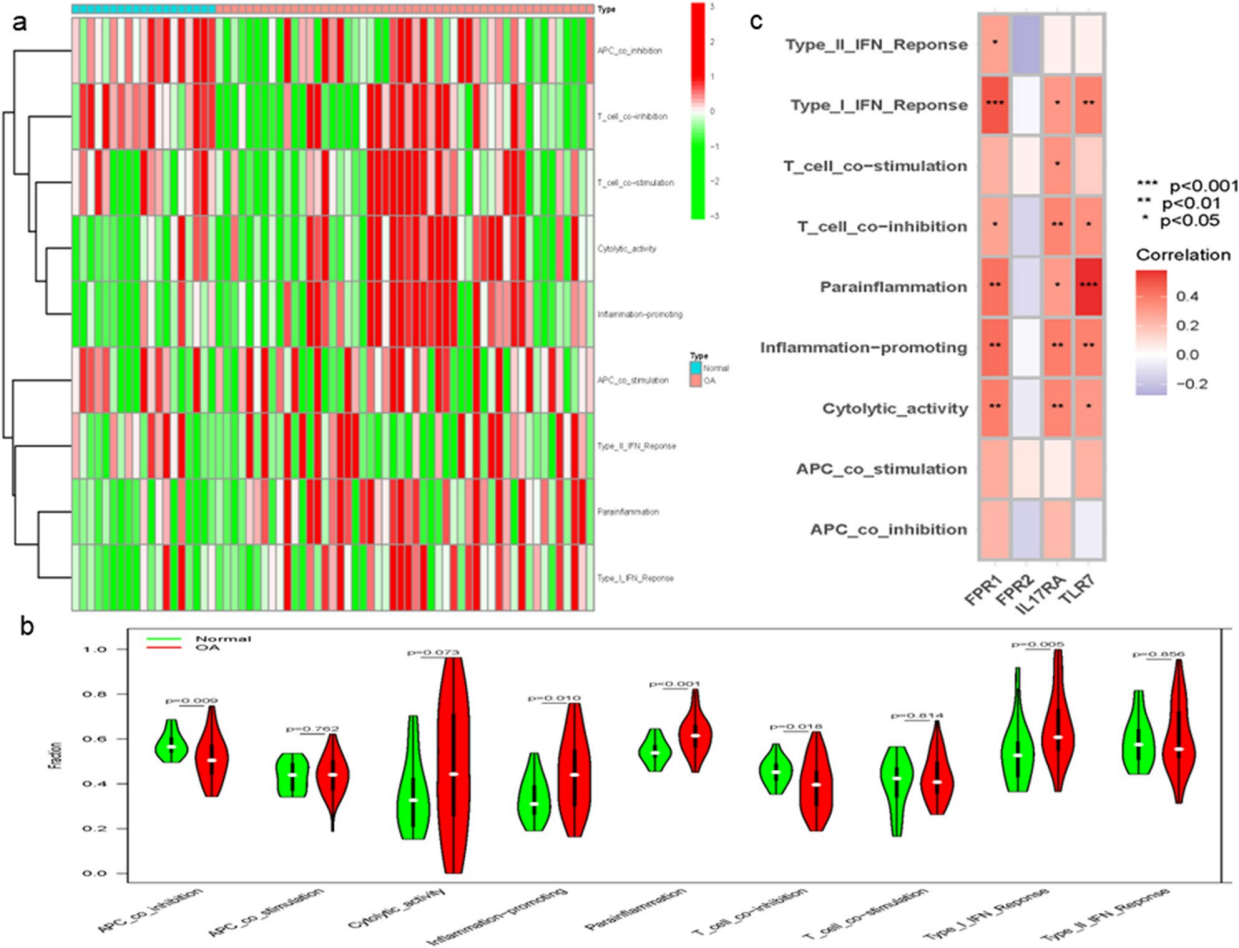
Analysis of immune functional differences associated with OA. Heatmap (**a**) and violin plot (**b**) showing the distribution of immune pathways in healthy control and OA samples. (**c**) The relationship between four hub genes and immune pathways.

The ssGSEA algorithm was used to determine the differences in immune cell function between OA and healthy controls. Figure 11 shows the distribution of immune pathways in samples.

### Candidate gene expression validations

**Figure 12:**
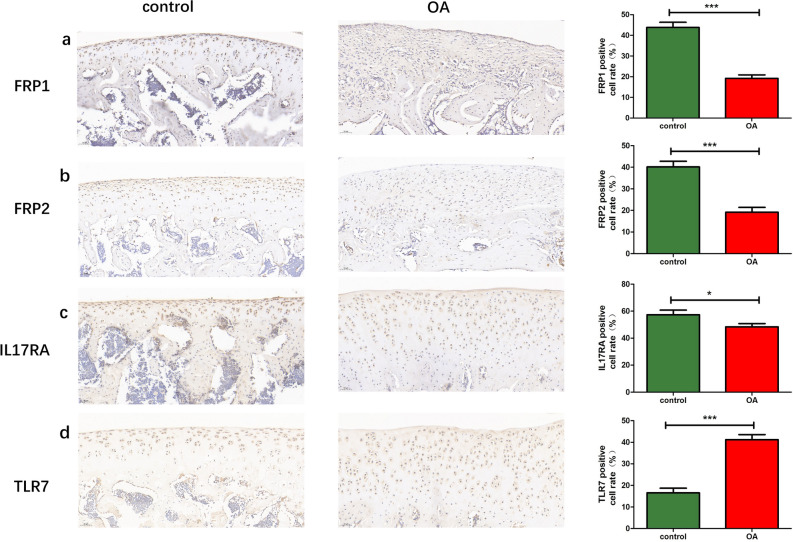
The expression levels of FPR1, FPR2, IL-17RA and TLR7 in the cartilage of OA rats changed. Immunostaining FPR1 (**a**), FPR2 (**b**), IL17RA (**c**), and TLR7 (**d**) were performed in control or OA model knee cartilage sections. Graphs showing comparisons of FPR1 (**a**, *P* < 0.0001), FPR2 (**b**, *P* < 0.0001), IL17RA (**c**, *P* < 0.05) or TLR7 (**d**, *P* < 0.0001) expression in knee joint sections of cartilage from 8 control or 10 OA models. Photos were magnified 200 times. Data are mean ± SD. Scale bar = 50 μm. ***P < 0.001; *P < 0.05.

We further studied the expressions of FPR1, FPR2, IL-17RA and TLR7 in the knee cartilage of SD rat OA model and control group by IHC. As shown in Fig. 12, FPR1, FPR2, and IL-17RA in OA cartilage were significantly down-regulated, while TLR7 was significantly up-regulated.

### qPCR validation of data

**Figure 13:**
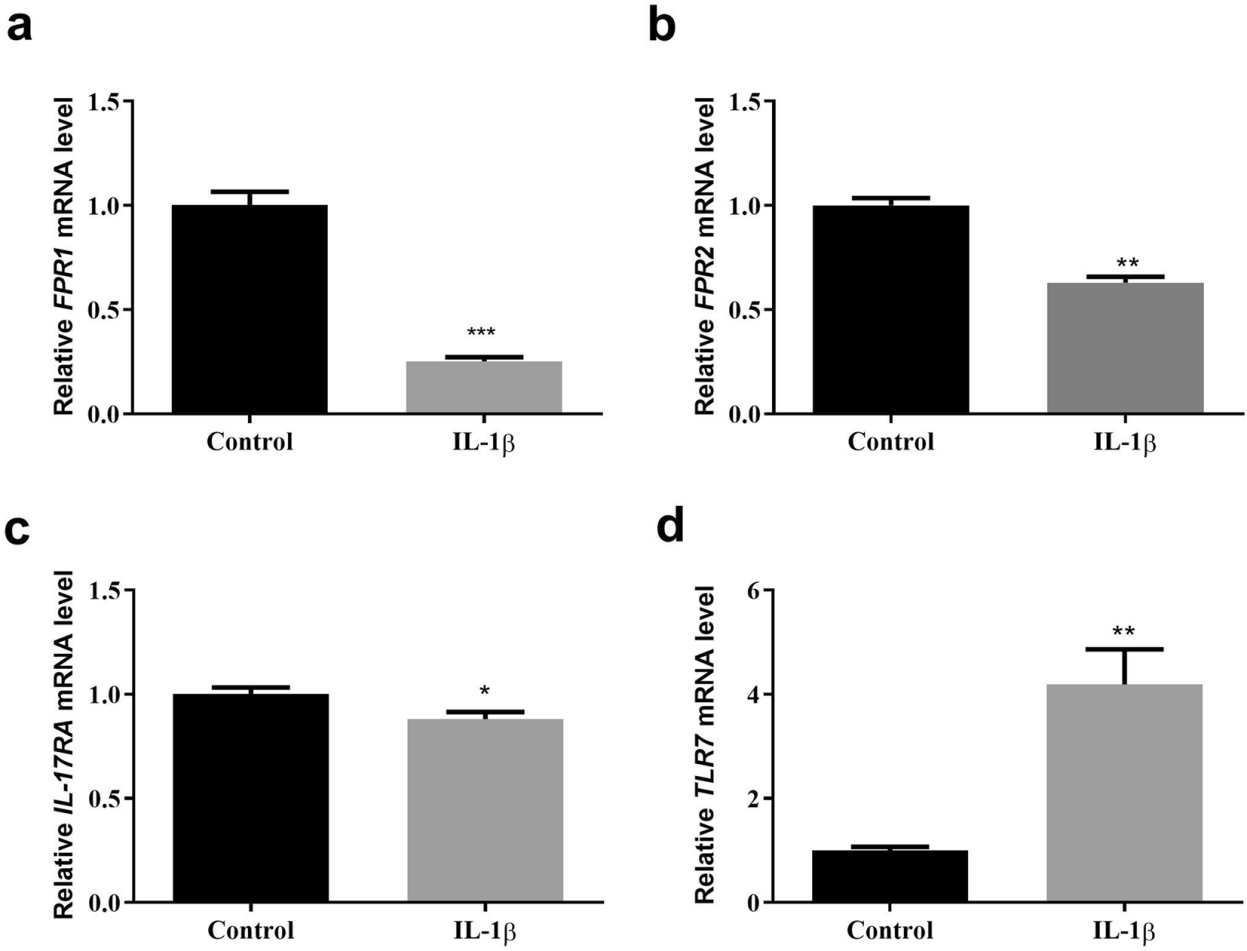
Validation of hub genes in rat chondrocytes. After IL-1β induced, RT-qPCR results detecting the mRNA expression of FPR1 (**a**), FPR2 (**b**), IL17RA (**c**), and TLR7 (**d**). Ns: not significant; *P < 0.05; **P < 0.01; ***P < 0.001.

To verify the bioinformatics results, qPCR experiments were conducted. The results revealed that the mRNA expression levels of FPR1, FPR2 and IL17RA were significantly lower while TLR7 was significantly higher in the IL-1β-induced group. This indicates that the results of data mining are reliable and have potential research value (Fig. 13).

### Knockdown of TLR7 reversed the effect of IL-1β on biomarkers of osteoarthritis in rat chondrocytes

**Figure 14:**
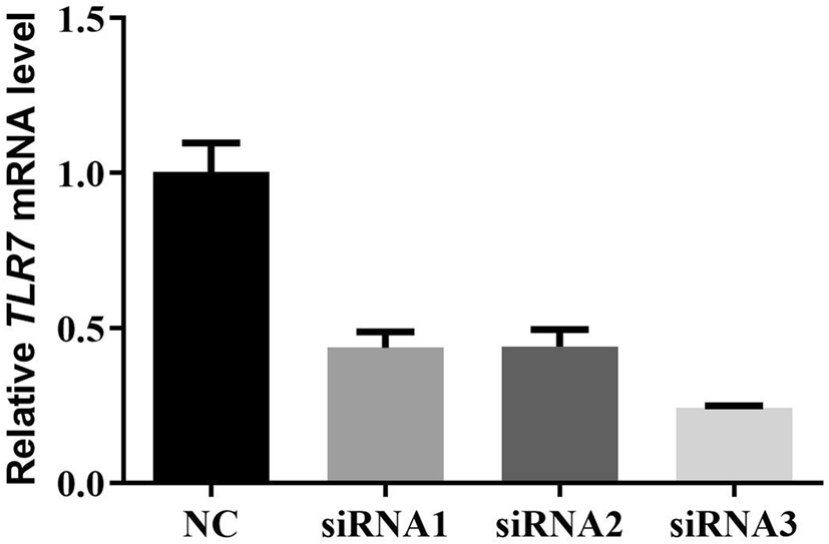
The effectiveness of siRNA TLR7 knockdown plasmids was verified by PCR experiment.

**Figure 15:**
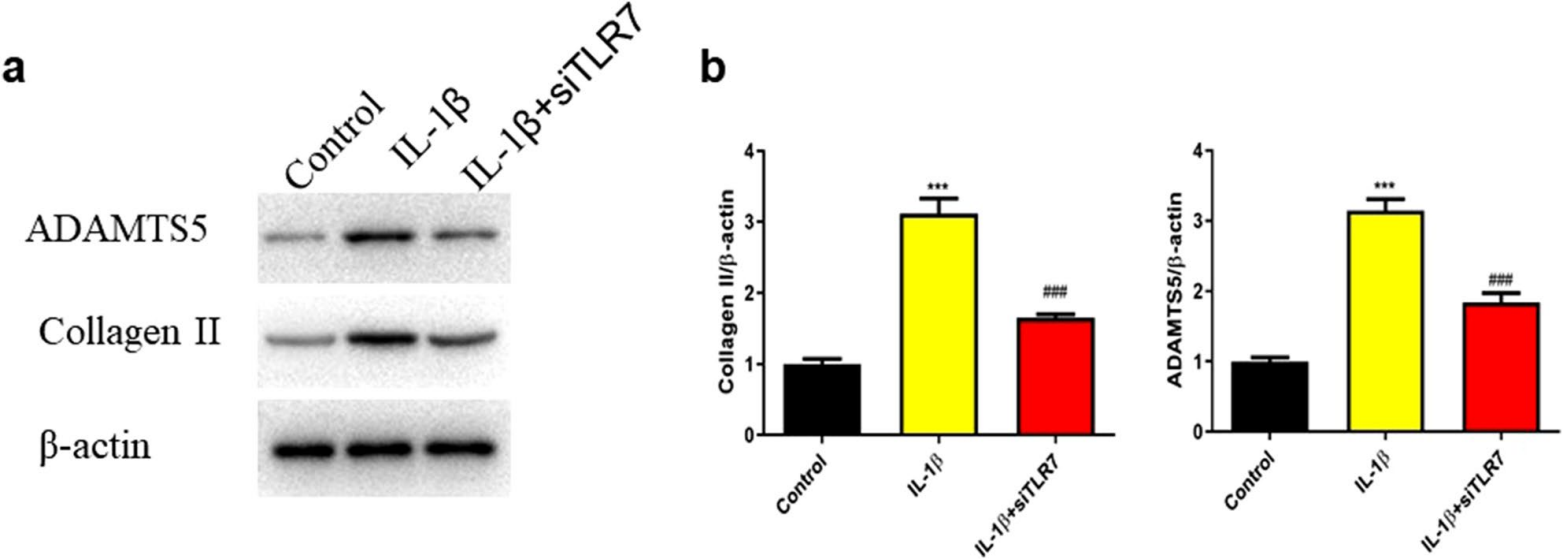
SiRNA TLR7 reversed collagen II and ADAMTS-5 degradation in IL-1β-stimulated chondrocytes. Cells were stimulated with IL-1β (10 ng/ml) for 48 h. The expression levels of collagen II and ADAMTS-5 were evaluated by Western blot (**a**) and quantification analysis (**b**). ***P < 0.001 versus the control group, ^###^*P* < 0.001 versus the IL-1β group.

According to our results (Fig. 13), the expression of TLR7 were upregulated in OA with significant difference, suggesting its effect in the pathological process of OA. In order to verify the potential effects of TLR7, after the effectiveness of the knockout plasmids has been verified, siRNA-TLR7 and siRNA-NC were transferred into rat chondrocytes (Fig. 14). Then, transfected rat chondrocytes were incubated with IL-1β (10 ng/ml, 48 h) for subsequent analysis. ADAMTS-5 and Collagen II were identified as the biomarkers of osteoarthritis^30^.The results showed that the expression of ADAMTS-5 and Collagen II proteins were upregulated by IL-1β and reversed after knockdown of TLR7(Fig. 15), suggesting that TLR7 may regulate IL-1β-induced chondrocyte damage and participate in the progression and development of OA.

## Discussion

ICD has received more and more attention in the area of both tumor and non-tumor diseases. Studies have confirmed that surface exposed calreticulin (CALR) and secreted ATP, annexin A1 (ANXA1), type I interferon and high mobility histone box 1 (HMGB1) are related to ICD^31,32^. The direct correlation between ICD and OA has not yet been reported. However, there are some similarities in the pathological process of ICD and OA, such as secreted ATP^33^, ANXA1^34^ and HMGB1^35^. We speculate that ICD may be related to OA, because the above ICD-related biomarkers have been found in OA. Therefore, to explore the relationship between ICD and OA may provide a new target for the diagnosis and treatment of OA. This study has identified four central genes that may be involved in OA and ICD-related pathologic processes. FPRs are a group of proteins in the G-protein-coupled receptors (GPCR) family that are found primarily in human neutrophils, but also in other species (horses, rabbits, and rodents) and other immune cell types (macrophages, monocytes, etc.)^36^. Human FPR1 is activated after infection and sterile stimulation, leading to immune cell responses^37^. It has been reported that FPR1 expressed higher in synovial membrane from experimental OA rat^38^. The IL-17 cytokine family is considered to be critically involved in the pathogenesis of OA^39^. IL-17 binds to IL-17 receptor A (IL-17RA)^40,41^ has been found highly expressed in immune cells^42,43^. However, the role of IL-17 induction may vary via cell type, focus should be placed on its various role in specific cell type or organ system^44,45^. IL-17RA is highly expressed in the cartilage and synovium of end-stage OA, suggesting its critical role in the pathophysiology of OA^46^. TLR7 derived from the TLR family has also been found to play an important role in immune-mediated inflammatory diseases^46,47^. Studies have showed that TLR7 signaling pathway was involved in immune cell activation and in triggering the secretion of chemokines and proinflammatory cytokines, which would further promote the development of inflammatory diseases, such as systemic lupus erythematosus^48^, rheumatoid arthritis^49^ and OA^50^. Moreover, TLR7 ligation can affect the expression of p21 and p-STAT3 to regulate tumor stromal inflammation^51^. It has been reported that TLRs can regulate the anabolic and catabolic pathways of OA and the apoptosis of chondrocytes, and the mechanism was related to the activation of innate immune response^52^. Importantly, in a post-traumatic mice OA model, lipopolysaccharide (LPS) was further used to induce an inflammatory response and TLR7 expression was found significantly upregulated^53^. Similarly, TLR7 expression was elevated in the synovial and blood samples of OA patients^54^. Emerging evidence has demonstrated that immune factors involve significantly in OA^55,56^. In our study, the expression levels of FPR1, FPR2, IL17RA, and TLR7 were significantly changed in the IL-1β induced rat chondrocytes as well as in the knee cartilage of SD rat OA model. And knockdown of TLR7 reversed collagen II and ADAMTS-5 degradation in IL-1β-stimulated chondrocytes. According to our findings, four hub genes (FPR1, FPR2, IL17RA, and TLR7) were ultimately identified as OA biomarkers associated with ICD.

Immunotherapies have shown therapeutic effects in animal models of OA^57^. Accordingly, OA patients may get benefit from immunotherapies aimed ICD as therapeutic targets. The experimental and clinical efficacy of ICD aimed drugs in the treatment of OA needs to be further confirmed. There are some limitations to this study. Firstly, there are only two selected datasets in this study, so we can only select limited data and OA patients, resulting in a limited number of genes available for final selection. Secondly, the ICD biomarkers related to OA identified in this study were only validated through Rat's animal and in vitro experiments, and further laboratory evidence is needed for validation. Thirdly, the ICD related genes selected in this study are sourced from a previous study, and more related genes may be discovered in the future (Supplementary Figures 1–7).

## Conclusion

We identified four hub genes (FPR1, FPR2, IL17RA, and TLR7) that were strongly associated with ICD in OA patients, which could distinguish OA patients from controls. This research may provide new immune related biomarkers for the diagnosis of OA and serve as a reference for disease treatment monitoring.

### Supplementary Information

Supplementary Information 1.

Supplementary Figures.

## Data Availability

All data generated or analysed during this study are included in this published article.

## Abbreviations

ICD: Immunogenic cell death
RCD: Regulated cell death
OA: Osteoarthritis
DEG: Differentially expressed genes
WGCNA: Weighted gene co-expression network analysis
LASSO: Least absolute shrinkage and selection operator
IHC: Immunohistochemistry
MCs: Mast cells
FPR1: Formylpeptide receptor 1
FPR2: Formylpeptide receptor 2
IL17RA: Interleukin-17 receptor A
TLR7: Toll-like receptor 7
AUC: Area under the curve
CALR: Calreticulin
ANXA1: Annexin A1
HMGB1: High mobility histone box 1
GPCR: G-protein-coupled receptors
IL-17RA: IL-17 receptor A
LPS: Lipopolysaccharide
ADAMTS: A Disintegrin and Metalloproteinase with Thrombospondin motifs
