# Supplementary material for: Identification and validation of FPR1, FPR2, IL17RA and TLR7 as immunogenic cell death related genes in osteoarthritis
Source: Sci Rep. 2023 Oct 6;13:16872. doi: 10.1038/s41598-023-43440-z (PMC10558501; doi:10.1038/s41598-023-43440-z)
Supplement: Supplementary file 1 — Supplementary Information 1. [file 41598_2023_43440_MOESM1_ESM.pdf]

R package version number

R “limma” 3.48.3

R “ggplot2” 3.3.5

R “ggrepel” 0.9.1

R “pheatmap” 1.0.12

R “venn” 1.11

R “dplyr” 1.1.2

R “WGCNA” 1.72.1

R "org.hs.eg. db" 3.16.0

R "clusterProfiler" 4.0.5

R "Enrichment Plot" 1.12.2

R “pROC” 1.18.0

R "GSVA" 1.40.1

R "vioplot" 0.4.0

R “reshape2” 1.4.4
