# Supplementary material for: Identification and validation of FPR1, FPR2, IL17RA and TLR7 as immunogenic cell death related genes in osteoarthritis
Source: Sci Rep. 2023 Oct 6;13:16872. doi: 10.1038/s41598-023-43440-z (PMC10558501; doi:10.1038/s41598-023-43440-z)
Supplement: Supplementary file 2 — Supplementary Figures. [file 41598_2023_43440_MOESM2_ESM.pdf]

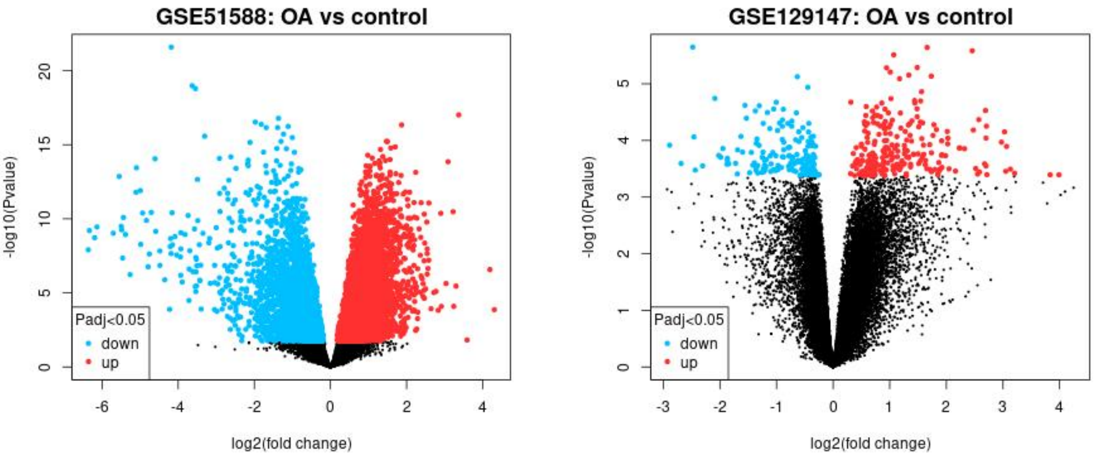

Supplementary Figure 1. The volcano plots of upregulated(red) and downregulated(blue) genes in GSE51588 and GSE129147.

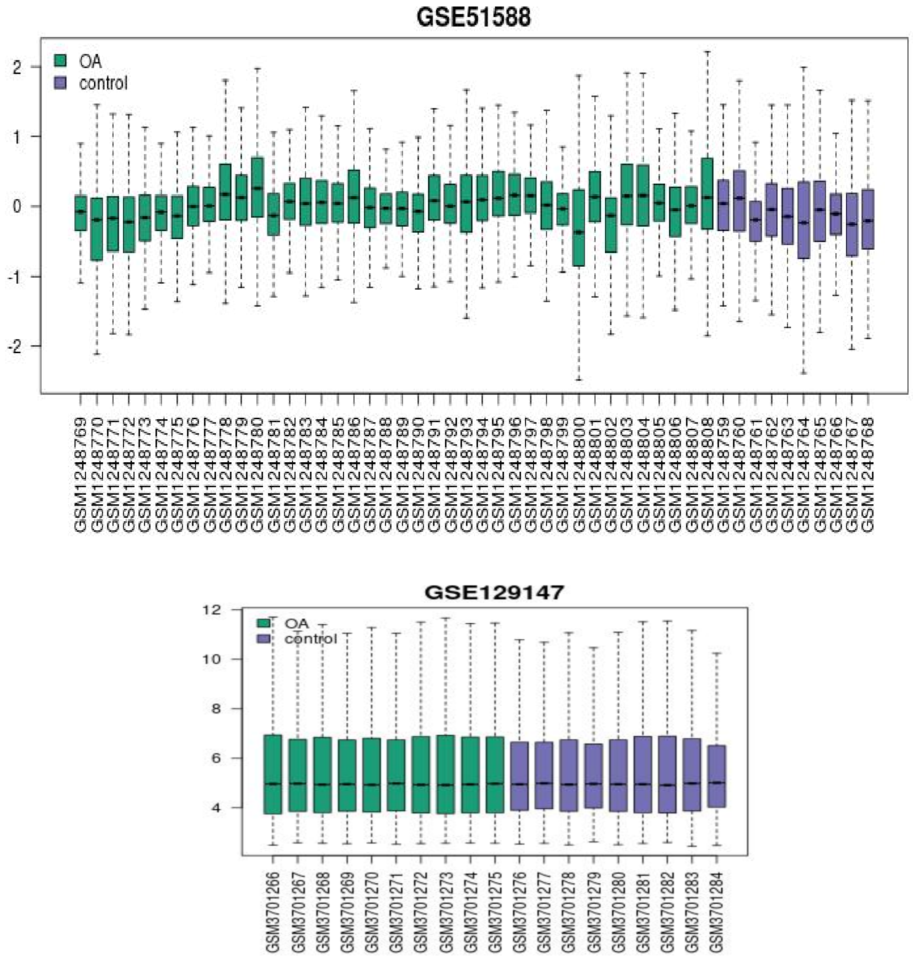

Supplementary Figure 2. Cross comparison of two groups of OA patients and normal controls. The green boxes refer to OA patients and the blue ones refer to normal controls.

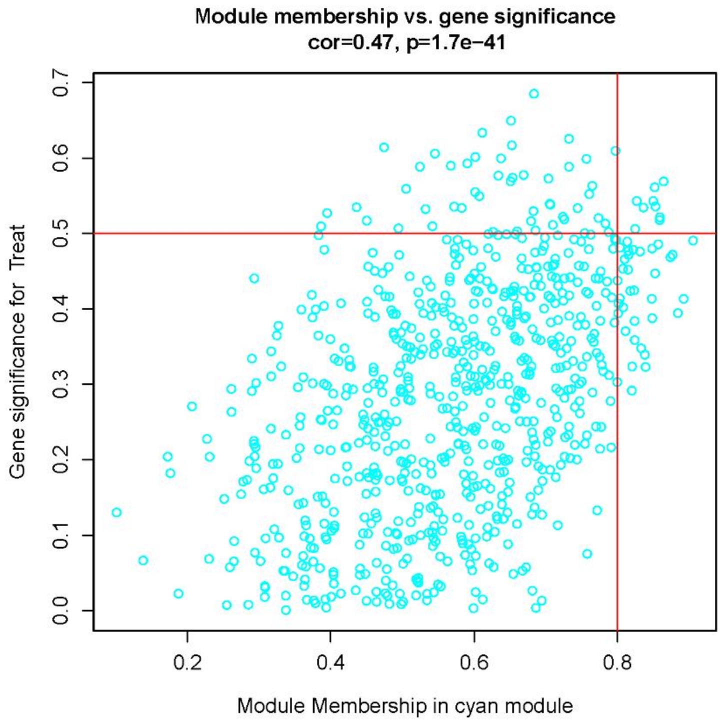

Supplementary Figure 3. A scatterplot of Gene Significance (GS) for weight vs. Module Membership (MM) in the module cyan.

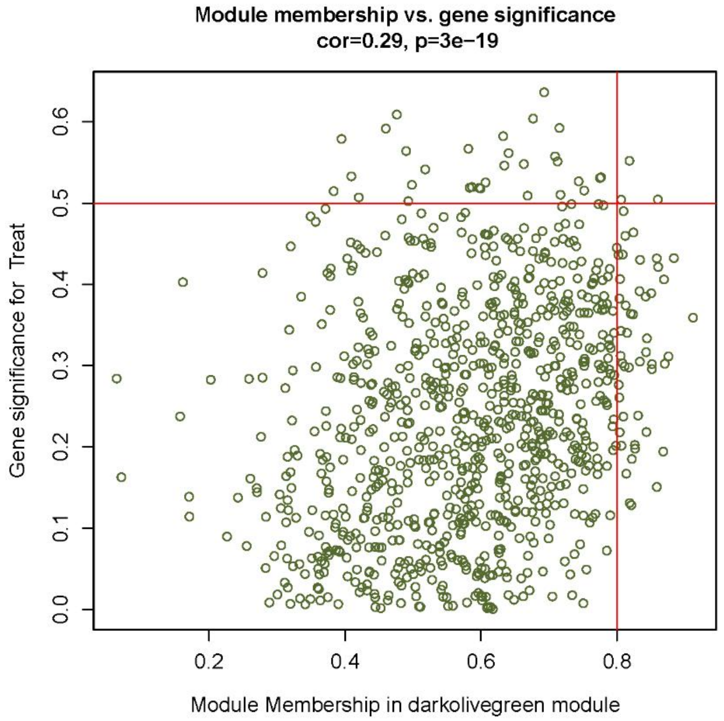

Supplementary Figure 4. A scatterplot of Gene Significance (GS) for weight vs. Module Membership (MM) in the module

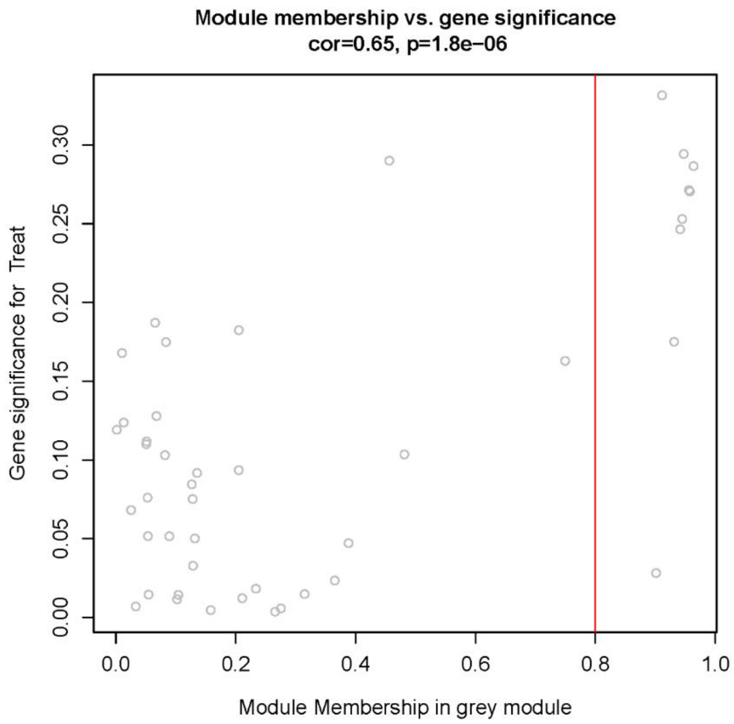

darkolivegreen

Supplementary Figure 5. A scatterplot of Gene Significance (GS) for weight vs. Module Membership (MM) in the module grey.

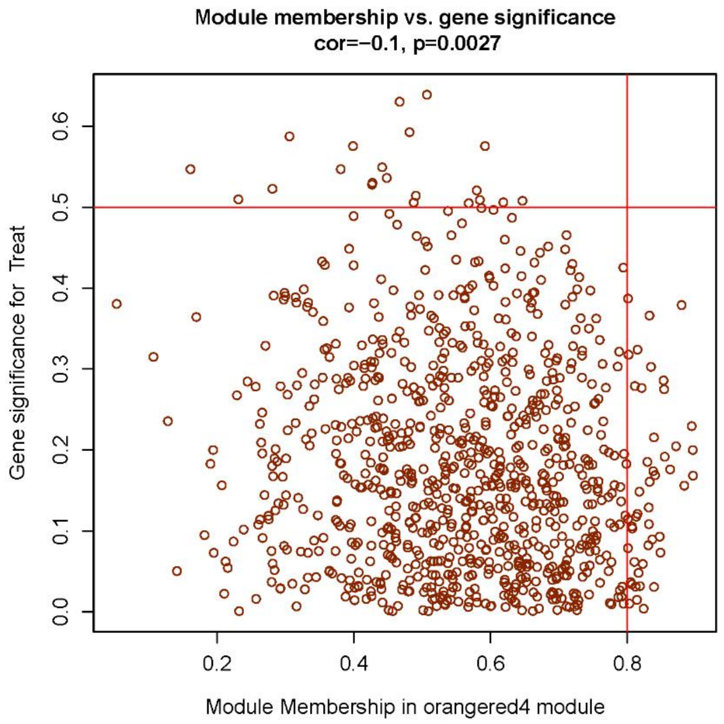

Supplementary Figure 6. A scatterplot of Gene Significance (GS) for weight vs. Module Membership (MM) in the module orangered4.

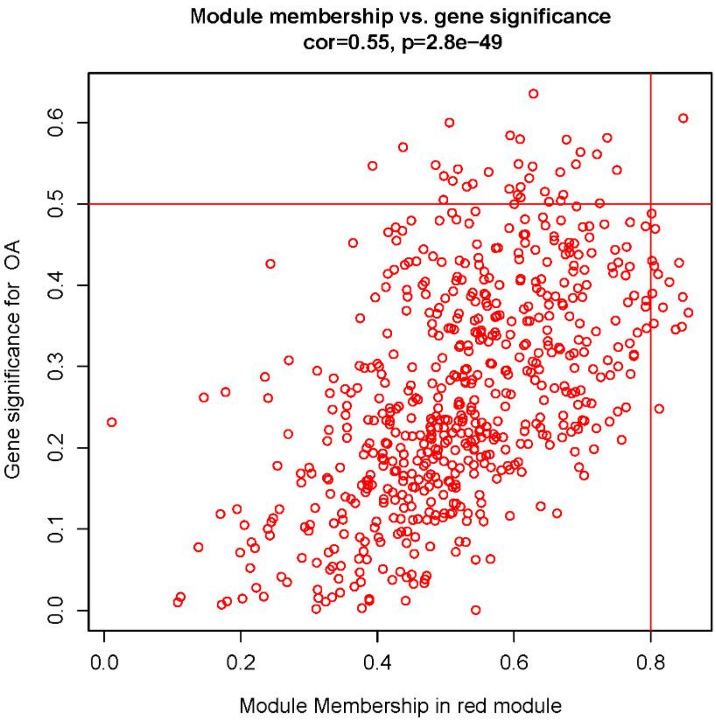

Supplementary Figure 7. A scatterplot of Gene Significance (GS) for weight vs. Module Membership (MM) in the module red.
